# Researchers' Mental Health and Quality of Life: A Protocol for Systematic Review and Meta‐Analysis

**DOI:** 10.1002/hsr2.72253

**Published:** 2026-05-14

**Authors:** Lavine Jordane Queiroz de Azevedo, Marcos de Moraes Sousa, Murilo Marques Costa, Lavínia Leal Cordeiro, Miguel Matos Torres, Suelen Marçal Nogueira, Priscilla Rayanne e Silva, Matias Noll

**Affiliations:** ^1^ Goiano Federal Institute, Campus Ceres Ceres Goiás Brazil; ^2^ Goiano Federal Institute, Campus Rio Verde Rio Verde Goiás Brazil; ^3^ Federal University of Goiás (UFG) Goiânia Goiás Brazil; ^4^ Evangelical University of Goiás, Campus Ceres Ceres Goiás Brazil; ^5^ Kent Business School University of Kent Canterbury Kent UK; ^6^ Goiano Federal Institute, Campus Urutaí Urutaí Goiás Brazil

**Keywords:** academic personnel, burnout, public policies, research & development, well‐being

## Abstract

**Background and Aims:**

Although several previous studies have examined the determinants of research productivity, the conditions under which researchers achieve high performance remain poorly understood. This protocol aims to lay the groundwork for a systematic review and meta‐analysis of the factors associated with researchers' mental health (RMH) and quality of life (RQoL) across public and private institutions, to provide future evidence to inform targeted policies.

**Methods:**

The protocol is registered with PROSPERO (CRD42025635600) following PRISMA‐P 2015 guidelines. Seven databases will be searched (CINAHL, LILACS, MEDLINE/PubMed, Scopus, Web of Science, EMBASE, and PsycArticles) for observational studies. Two independent reviewers will screen titles/abstracts and full texts, with discrepancies resolved by a third reviewer. Methodological quality (GRADE), risk of bias (Downs and Black scale), and interrater reliability (Cohen's *κ*) will be assessed. Descriptive synthesis and meta‐analysis (when appropriate) will explore subgroup analyses by sex/gender, academic role, and geographic region. Heterogeneity will be assessed using Cochran's *Q* and *I* ^2^ statistics.

**Results:**

This is a study protocol, therefore no results are available at this stage. The review will systematically synthesize the available evidence on the association between RQoL and RMH and, where possible, provides combined effect estimates through meta‐analysis.

**Conclusion:**

The systematic review and meta‐analysis will highlight challenges in the academic environment and provide evidence to inform the development of more sustainable working conditions.

**Trial Registration:**

PROSPERO (CRD42025635600).

## 1 Introduction

Researchers, including scientists, graduate students, and postdoctoral researchers, constitute a very distinct group of professionals, who have important roles in analyzing, generating, and disseminating knowledge providing support for policy [1]. These professionals are key to advancing science and technology, testing and validating new ideas and theories, analyzing data, exploring and developing innovative techniques [2, 3]. This work involves individual, interpersonal, and systemic factors that may influence researchers' mental health (RMH) and quality of life (RQoL) [4, 5].

Initiatives such as Sustainable Development Goal 3 (SDG 3), proposed by the United Nations, have become fundamental because they aim to ensure healthy lives and promote well‐being for all, including those dedicated to scientific research, across all areas and stages of life [6, 7]. The World Health Organization (WHO) defines RQoL as individuals' perceptions of their social position in the sociocultural context and their goals, expectations, standards and concerns [8]. Sociodemographic factors, such as sex, gender, and marital status [9, 10, 11], may be associated with RQoL and, consequently, affect RMH. Conditions of work, as aspects of work, are also associated with workload, production expectations established by academic systems, and institutional support, among other factors, which are common among academia [12, 13, 14, 15]. Symptoms of depression, burnout, anxiety, and stress have been frequently reported, contributing to a decline in quality of life [16]. For example, among undergraduate courses, high work demands and RQoL are often associated with significant negative effects on physical health, psychological well‐being, social relationships, and the work environment [17]. Furthermore, women working in research environments, such as postgraduate programs, report higher levels of an increasing perceived stress and are at particular risk of developing RMH problems [11, 18, 19, 20].^1^

Guthrie and colleagues reported that more than 40% of postgraduate students in the United Kingdom experienced symptoms of depression or emotional difficulties related to stress [21]. A systematic review by Nicholls and colleagues identified structural and organizational factors, such as job insecurity, limited family‐friendly policies, and stringent funding and promotion requirements, as major contributors to stress among researchers [5]. Sustained pressure to maintain high levels of productivity was also associated with an increased risk of mental and physical health problems in academic populations [22].

Although some studies address RMH [10, 23, 24] and RQoL [25, 26] separately, there is a significant gap in understanding the factors that simultaneously influence this relationship. This systematic review and meta‐analysis takes an integrated approach to mental health and quality of life, treating them as interdependent dimensions that reflect the complex nature of researchers' working lives. To guide this investigation, the following research question was established: “Which factors are associated with RMH and RQoL among researchers working in public and private institutions?” This protocol aims to lay the groundwork for a systematic review and meta‐analysis of the factors associated with RMH and RQoL across public and private institutions, to provide future evidence to inform targeted policies This review is grounded in the following theoretical hypotheses: (i) sociodemographic characteristics such as sex, race, and minority status may be associated with poorer RMH and RQoL; (ii) high work demands, job insecurity and productivity pressure may negatively affect RMH and RQoL; (iii) low institutional support may predict worse RMH and RQoL outcomes; and (iv) symptoms of depression, anxiety and stress may mediate the relationship between working conditions and RQoL. These hypotheses do not predetermine results but provide a conceptual foundation and highlight the complexity of researchers' working lives.

## 2 Methods

### 2.1 Protocol and Registration

The protocol follows the Preferred Reporting Items for Systematic Review and Meta‐Analysis Protocols 2015 (PRISMA‐P) guidelines, which provide a clear roadmap for conducting systematic reviews (Supporting Information S1: File 1) [27, 28, 29]. This protocol was registered in the International Prospective Register of Systematic Reviews (PROSPERO) under the number CRD42025635600, which is a prospective international registry for systematic review protocols [27, 28]. This registry was performed in the initial stage of protocol construction, helping to minimize duplications and improve quality [30]. The final article was directed to the registration information [31, 32]. Any changes to this protocol during the study will be documented in PROSPERO and in the final manuscript [33]. Given that this study consists of a systematic review and meta‐analysis protocol based exclusively on published data, ethical approval and informed consent are not required.

### 2.2 Research Strategy and Databases

This systematic review is structured using the PECOS strategy (population, exposure, comparison, outcome, and study design) [34, 35]. This mechanism directs the search strategy, using keywords and related descriptors to search databases [36] and is defined on the basis of the research problem addressed by the systematic review. The strategy is composed of three blocks related to the following guiding terms: P (Population) “professionals whose work involves the systematic conduct of scientific research, regardless of their employment status or the type of institution in which they work, in public or private institutions,” E (Exposure) “factors associated with working conditions, institutional practices, occupational demands, organizational characteristics, or psychosocial aspects that may influence the researcher's well‐being,” C (Comparison) “groups of researchers not exposed or less exposed to these same factors,” O (Outcome) “outcomes related to RMH and RQoL,” and S (Study design) “delimitation in observational studies.” The synonyms of these terms and their index values are used in controlled vocabulary databases to identify relevant studies, with each descriptor connected by the Boolean operator “OR” and the blocks combined via the operator “AND” presents the blocks and descriptors included in the electronic search strategy.

The sampling strategy refers to the ability to identify all possible original articles, selectivity refers to relevant articles, and objectivity refers to maintaining a specific focus on a subject of interest [37]. The database searches were performed via the following fields: title, abstract and keywords. For this comprehensive research, databases with international reach encompassing multidisciplinary content were selected to ensure a complete global view. The following databases are consulted: Cumulative Index to Nursing and Allied Health Literature (CINAHL) via the EBSCOhost interface, Latin American and Caribbean Health Science Literature (LILACS) via the Virtual Health Library (BVS), MEDLINE/PubMed via the National Library of Medicine (NLM) interface, Scopus, Web of Science (WoS) Core Collection, EMBASE via the native interface and PsycArticles via the APA PsycNet interface. A comprehensive approach to the subject is ensured, with varying depths across the consulted databases.

Reviewer R1 will perform the extraction from the databases in April 2026, and the search strategy for each database will be adapted to its unique characteristics and respective search interfaces (Supporting Information S2: File 2). Data extraction from the databases follows the guidelines established in the Preferred Reporting Items for Systematic Reviews and Meta‐Analyses‐Search (PRISMA‐S) checklist for metadata extraction, which lists 27 items that must be included when reporting a systematic review [32]. The PRISMA‐S covers topics such as the specifics of the selected databases, the search strategy (including the definitions of limits, restrictions, and filters), and the documentation of returned records and duplicates [38, 39]. The guidelines of the Peer Review of Electronic Search Strategies (PRESS) for systematic reviews (SRs) are used to maximize the effectiveness and reach of the search strategies. In the evidence synthesis process, it is at the beginning of the project, before conducting the research, and the prepublication phase [40].

### 2.3 Eligibility Criteria

Studies were included based on the following criteria:

#### 2.3.1 Inclusion Criteria

(i1) Original articles published after peer review.

(i2) Studies published without restriction on publication date [41, 42].

(i3) Studies in which the sample consists of researchers, defined as professionals or students who perform scientific research activities (conducting studies, publishing scientific outputs, applying for funding) [5].

(i4) Studies that evaluated the factors associated [41] with both outcomes: (I) RMH, including concepts such as depression, anxiety, burnout, stress, psychological distress, or related indicators, assessed by validated instruments (classical or work‐specific), and (II) RQoL, defined by multidimensional frameworks, such as those proposed by the WHO, or conceptually equivalent validated models.; and no language restrictions to minimize bias.

(i5) No language restrictions will be applied to avoid language bias.

(i6) Quantitative observational designs (cross‐sectional, case‐control, cohort) and mixed methods [43].

(i7) Studies will not be excluded based on risk of bias classification. Instead, risk‐of‐bias assessments will inform the narrative synthesis and, where appropriate, sensitivity analyses to assess the robustness of the findings.

#### 2.3.2 Exclusion Criteria

(e1) Duplicate articles were published on the same topic by the same authors [44, 45]. In these cases, the most comprehensive articles were considered.

(e2) Studies containing retraction records [46].

(e3) Studies that simultaneously approached samples with professionals who work with research and no researchers, unless the data have been reported separately or can be calculated from the data provided.

(e4) Studies not available in full in the databases researched, and those that could not be accessed even after attempts to contact the authors [47].

(e5) Articles written in restricted languages that cannot be translated correctly if the following measures are unsuccessful: translation using artificial intelligence tools, with independent review by bilingual researchers and, when necessary, contact with authors for clarification [42, 44].

(e6) Studies with incomplete data or literature review articles, opinion articles, case reports, comments, editorials, dissertations, and similar reviews.

We exclude studies that do not comply with the criteria above and use Scite (https://scite.ai/home) to check the validity of the evidence.

### 2.4 Process of Review and Selection of Articles

After completing the database searches using predefined strategies, the results will be imported in RIS format into Rayyan software, a web‐based platform designed to support systematic review screening and selection processes [48]. This tool facilitates duplicate removal and the initial screening of titles and abstracts through a semi‐automated process with high usability. Screening is performed independently and in pairs (R1 and R2), with any disagreements resolved by a third reviewer (R3), in accordance with the predefined eligibility criteria [29, 42]. Full‐text articles should be reviewed independently by R1 and R2, with conflicts resolved by R3. To minimize bias, the entire review process follows a blinded, parallel, and independent protocol for screening, study selection, data extraction, and quality assessment [49].

**Figure 1:**
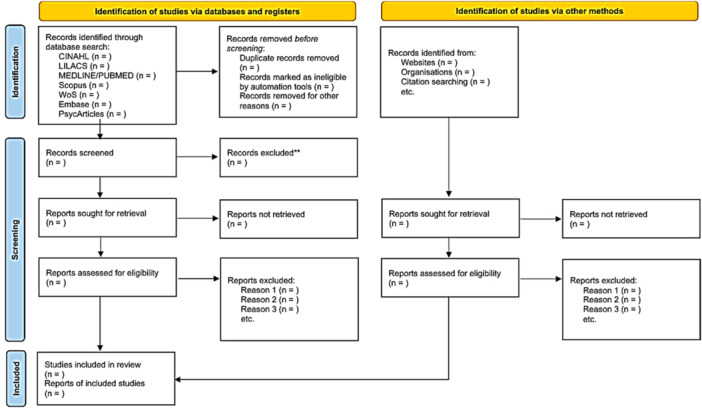
Template of the PRISMA 2020 flow diagram to be completed after the study selection process.

The studies identified through the search strategy will be screened and selected according to the eligibility criteria established in this protocol. Additional records may be identified through manual searches of the reference lists of the included studies. After the selection process is complete, the final set of eligible articles will be included in the systematic review. The study selection process will be reported using a PRISMA 2020 flow diagram. An illustrative flowchart is presented in Figure 1 [50].

Statistical analyses will be performed using the Statistical Package for the Social Sciences (SPSS), version 26 (IBM Corp., Armonk, NY). To ensure methodological rigor and transparency, interrater reliability will be assessed. The percentage of agreement between reviewers will be calculated, followed by Cohen's *κ* coefficient, which adjusts for chance agreement, providing a more robust and reliable measure of consistency throughout the screening process [51, 52, 53, 54].^2^

### 2.5 Data Extraction, Data Synthesis and Analysis

A spreadsheet should be used to extract data from quantitative and mixed‐methods studies. The extracted variables include the first author, year and country of publication, study design, title, objective, population characteristics and academic role, sample size, age range, data collection instruments, outcomes assessed, associated factors, main findings, effect size measures with corresponding 95% confidence intervals (CI), and statistical tests applied and reported research gaps (Supporting Information S3: File 3). Data extraction will be carried out independently by two reviewers (R1 and R2) [55], with any discrepancies resolved by consensus with a third reviewer (R3). When additional relevant information was identified during the extraction process, the data extraction tool will be updated accordingly, and all modifications will be documented in the final review report. The planned statistical reporting will follow established recommendations for clarity, transparency, and interpretability of clinical research data, particularly the reporting principles proposed by Assel and colleagues, prioritizing effect sizes, CI, and appropriate interpretation of *p* values [56].

The data should be summarized descriptively, and when appropriate, prespecified subgroup analyses will include sex/gender, academic role (students, faculty, researchers), and geographic region to explore patterns and differences in the relationships between work conditions, mental health, and quality of life. When applicable, study results will be reported using effect sizes (odds ratios, mean differences) with corresponding 95% CI and *p* values, avoiding an isolated presentation of statistical significance [57]. *p* values are reported as *p* < 0.001 for values below 0.001, with three decimal places for values between 0.001 and 0.01, and two decimal places for values ≥ 0.01. *p* values > 0.99 were reported as *p* > 0.99.

A meta‐analysis may be considered if the collected data can be appropriately standardized and grouped, allowing for statistical synthesis that integrates results from prior studies and provides a comprehensive overview [58, 59, 60]. This is performed only when the data are sufficiently consistent and comparable across studies. All the statistical tests will be two‐sided. Statistical interpretation will prioritize effect sizes and corresponding 95% CI, while *p* values will be reported only as complementary information following recommended reporting standards [61]. Descriptive and interrater agreement analyses are performed using SPSS version 26 (IBM Corp., Armonk, NY, USA), while meta‐analytical procedures—when applicable—use STATA version 18.5 (StataCorp, College Station, TX, USA) [62]. Reporting statistical methods and results will follow SAMPL recommendations [63, 64].

### 2.6 Methodological Quality and Risk of Bias Assessment

The included articles will be evaluated based on the quality of their evidence, following the recommendations of the Grading of Recommendations, Assessment, Development, and Evaluations (GRADE) (https://www.gradepro.org/) [65]. This system allows the assessment of certainty in a set of evidence and uses two levels, serious and very serious, to lower the quality in a single domain. The GRADEpro GDT software was used for this purpose [66, 67]. The GRADE classifications are incorporated directly into the summary of results, guiding the interpretive strength of the evidence and the robustness of the conclusions presented, without constituting an automatic criterion for the exclusion of studies.

The risk of bias in individual studies is assessed independently by two reviewers (R1 and R2), with disagreements resolved by a third reviewer (R3). For quantitative observational studies, the Downs and Black scale was used, comprising 27 items that assess methodological quality, external validity, bias, control of confounding factors, and statistical power [68]. The final score is expressed as a percentage of the maximum possible score, classifying studies as low risk of bias (> 70%) or a high risk of bias (< 70%) [49, 68]. The assessment must be carried out by two independent reviewers (R1 and R2), and any discrepancies resolved by a third senior reviewer (R3). This classification is used to qualify the interpretation of the findings in the narrative synthesis and, when applicable, to weigh the relative weight of the studies in the quantitative analysis, ensuring that evidence of greater methodological rigor contributed more consistently to the study's conclusions. Studies will not be excluded based on risk of bias classification. Instead, risk of bias assessments will inform the narrative synthesis and, where appropriate, sensitivity analyses to explore the robustness of the findings.

The feasibility of conducting a meta‐analysis to assess the association between RMH and RQoL will be evaluated if sufficient, comparable data are available. Random‐effects meta‐analyses will be conducted to account for potential heterogeneity between studies [61]. Statistical heterogeneity will be assessed using Cochran's *Q* test [69] and the *I* ^2^ statistic [70], which estimates the proportion of variability attributable to true heterogeneity. *I* ^2^ values will be interpreted as low (0%–40%), moderate (30%–60%), substantial (50%–90%), or considerable (75%–100%) [71]. Publication bias will be assessed using funnel plots and Egger's regression test [72]. All analyses will be performed using STATA version 19.0 (StataCorp, College Station, TX, USA).

### 2.7 Review and Training

Reviewers R1 and R2 will undergo training in using instruments for assessing methodological quality, risk of bias, and analyses via Rayyan software, which will be used to perform the systematic review. The authors responsible will also receive training to improve their evaluation of article eligibility and their use of inclusion and exclusion criteria [43]. For this purpose, the training consists of analyzing 50 articles and considers their respective titles and abstracts [44, 47, 73, 74]. These articles will be used exclusively for training and preparation for the systematic review proposed in this study protocol.

## 3 Discussion

RMH and RQoL have been reported in the literature as being influenced by structural aspects of the academic environment. The ongoing pressure to increase productivity remains as one of the most significant stressors. This pressure reflects performance‐based systems that prioritize production metrics at the expense of sustainable working conditions [75, 76]. Also, competitiveness in academic institutions seems intensified by limited funding, excessive workloads, and increasing professional responsibilities. Previous studies suggest that these conditions may contribute to chronic stress and emotional exhaustion at different career stages [5].

Early‐career researchers seem particularly susceptible to these dynamics. Financial insecurity and limited job stability are also described as relevant factors associated with RMH [19]. Evidence indicates that precarious employment may constitute a central determinant of compromised well‐being in academia [76]. Limited access to research infrastructure, mental health services, and structured wellness programs increases vulnerability to stress‐related conditions [24, 77]. The literature suggests that the absence of such support reflects systemic gaps rather than individual limitations [5]. Supportive institutional practices have been associated with improved professional functioning and well‐being outcomes [78, 79].

This systematic review protocol builds upon and extends previous reviews, such as those by Nicholls et al. [5] and Satinsky et al. [9], by reinforcing an integrated perspective that jointly examines RMH and RQoL. This approach goes beyond isolated outcomes and highlights the interdependence between working conditions, psychological well‐being, and broader domains of life. These considerations align with international debates that conceptualize researcher mental health as a global and systemic problem shaped by structural academic pressure rather than individual vulnerability alone [80, 81, 82]. Such discussions also emphasize the importance of strengthening mentorship, fostering persistence, and normalizing learning from failure as part of a healthier academic culture [83]. Building on this systemic perspective, the review is expected to contribute to ongoing discussions on institutional and policy‐level strategies. Regulating workload, professional stability initiatives, and structured support systems may contribute to more sustainable research environments and long‐term academic well‐being.

## Author Contributions

**Lavine Jordane Queiroz de Azevedo:** conceptualization, methodology, validation, data curation, writing – original draft, writing – review and editing, visualization, supervision, and project administration. **Marcos de Moraes Sousa:** conceptualization, methodology, data curation, writing – original draft, writing – review and editing, visualization, and project administration. **Murilo Marques Costa:** validation, writing – original draft, writing – review and editing, and visualization. **Suelen Marçal Nogueira:** conceptualization, writing – original draft, writing – review and editing, and visualization. **Lavínia Leal Cordeiro:** validation and writing – original draft. **Miguel Matos Torres:** writing – original draft, writing – review, and visualization. **Priscilla Rayanne E. Silva:** conceptualization, writing – original draft, and supervision. **Matias Noll:** conceptualization, methodology, validation, data curation, writing – original draft, writing – review and editing, visualization, supervision, and project administration. All authors have read and approved the final version of the manuscript. Lavine Jordane Queiroz de Azevedo had full access to all the data in this study and takes complete responsibility for the integrity of the data and the accuracy of the data analysis.

## Conflicts of Interest

The authors declare no conflicts of interest.

## Transparency Statement

The lead author Lavine Jordane Queiroz de Azevedo affirms that this manuscript is an honest, accurate, and transparent account of the study being reported; that no important aspects of the study have been omitted; and that any discrepancies from the study as planned (and, if relevant, registered) have been explained.

## Supporting information

Supplementary_file_S1_PRISMAP_R2.docx.

Supplementary_file_S2_table_strings_in_databases_R2.docx.

Supplementary_file_S3_table_included_in_the_SR_R2.docx.

## Data Availability

Data sharing is not applicable to this article as no new data were generated or analyzed in this protocol. All the data included in the future systematic review will be extracted from published studies available in the public domain.

## Endnotes

1 Satinsky et al. [9] and Trang et al. [20] compared different groups of researchers and reported that symptoms of anxiety, depression, and/or stress are more pronounced among specific populations, such as women, Black individuals, and members of the LGBTQ+ community.

2 The formula for calculating the *κ* coefficient is presented below:
$k=\frac{\mathrm{Po}-\mathrm{Pe}}{1-\mathrm{Pe}}.$
In this equation, the variable *Po* represents the observed proportion of agreement between reviewers, whereas *Pe* indicates the proportion of agreement expected by chance. The value of *κ* theoretically ranges between −1 and 1. When reviewers make the same decision for all items, *κ* reaches a maximum value of 1. The common interpretations of this value are as follows:

- *κ* = 1→ indicates perfect agreement.
- *κ* > 0→indicates agreement beyond what would be expected by chance.
- *κ* = 0→ indicates agreement equal to random agreement.
- *κ* < 0→indicates disagreement beyond what would be expected by chance.

## References

[1] J. L. Ballesteros‐Rodríguez , P. De Saá‐Pérez , N. García‐Carbonell , F. Martín‐Alcázar , and G. Sánchez‐Gardey , “Exploring the Determinants of Scientific Productivity: A Proposed Typology of Researchers,” Journal of Intellectual Capital 23, no. 2 (February 2022): 195–221, 10.1108/JIC-07-2019-0178.

[2] T. P. Vasileva , M. A. Yakushin , E. V. Makarova , P. I. Reshetnikova , G. E. Shukurlaeva , and M. D. Vasilev , “The Russian Scientists' Quality of Life and Cognitive Status,” European Journal of Translational Myology 31, no. 2 (June 2021), 10.4081/ejtm.2021.9744. PMC8274221 34148336

[3] D. R. Ciocca and G. Delgado , “The Reality of Scientific Research in Latin America; an Insider's Perspective,” Cell Stress and Chaperones 22, no. 6 (November 2017): 847–852, 10.1007/s12192-017-0815-8. 28584930 PMC5655372

[4] E. Cage , M. Stock , A. Sharpington , E. Pitman , and R. Batchelor , “Barriers to Accessing Support for Mental Health Issues at University,” Studies in Higher Education 45, no. 8 (August 2020): 1637–1649, 10.1080/03075079.2018.1544237.

[5] H. Nicholls , M. Nicholls , S. Tekin , D. Lamb , and J. Billings , “The Impact of Working in Academia on Researchers' Mental Health and Well‐Being: A Systematic Review and Qualitative Meta‐Synthesis,” PLoS One 17, no. 5 (May 2022): e0268890, 10.1371/journal.pone.0268890. 35613147 PMC9132292

[6] A. Madill , P. Bhola , E. Colucci , K. Croucher , A. Evans , and R. Graber , “How Can We Mainstream Mental Health in Research Engaging the Range of Sustainable Development Goals? A Theory of Change,” PLOS Global Public Health 2, no. 8 (August 2022): e0000837, 10.1371/journal.pgph.0000837. 36962779 PMC10022371

[7] J. Heymann and A. Sprague , “Meeting the UN Sustainable Development Goals for Mental Health: Why Greater Prioritization and Adequately Tracking Progress Are Critical,” World Psychiatry 22, no. 2 (June 2023): 325–326, 10.1002/wps.21090. 37159374 PMC10168142

[8] WHOQOL Group , “The World Health Organization Quality of Life Assessment (WHOQOL): Position Paper From the World Health Organization,” *Social Science and Medicine* 41, no. 10 (November 1995): 1403–1409, 10.1016/0277-9536(95)00112-K. 8560308

[9] E. N. Satinsky , T. Kimura , M. V. Kiang , et al., “Systematic Review and Meta‐Analysis of Depression, Anxiety, and Suicidal Ideation Among Ph.D. Students,” Scientific Reports 11, no. 1 (July 2021): 14370, 10.1038/s41598-021-93687-7. 34257319 PMC8277873

[10] S. Sokratous , G. Alexandrou , R. Zavrou , and M. Karanikola , “Mental Health Status and Stressful Life Events Among Postgraduate Nursing Students in Cyprus: A Cross‐Sectional Descriptive Correlational Study,” BMC Nursing 22, no. 1 (August 2023): 294, 10.1186/s12912-023-01463-x. 37644498 PMC10466854

[11] T. M. Evans , L. Bira , J. B. Gastelum , L. T. Weiss , and N. L. Vanderford , “Evidence for a Mental Health Crisis in Graduate Education,” Nature Biotechnology 36, no. 3 (March 2018): 282–284, 10.1038/nbt.4089. 29509732

[12] J. Stubb , K. Pyhältö , and K. Lonka , “Balancing Between Inspiration and Exhaustion: PhD Students' Experienced Socio‐Psychological Well‐Being,” Studies in Continuing Education 33, no. 1 (March 2011): 33–50, 10.1080/0158037X.2010.515572.

[13] E. Waight and A. Giordano , “Doctoral Students' Access to Non‐Academic Support for Mental Health,” Journal of Higher Education Policy and Management 40, no. 4 (July 2018): 390–412, 10.1080/1360080X.2018.1478613.

[14] X. Wang , C. Wang , and J. Wang , “Towards the Contributing Factors for Stress Confronting Chinese PhD Students,” International Journal of Qualitative Studies on Health and Well‐Being 14, no. 1 (January 2019), 10.1080/17482631.2019.1598722. PMC6495111 31021309

[15] V. Gewin , “Pandemic Burnout Is Rampant in Academia,” Nature 591, no. 7850 (March 2021): 489–491, 10.1038/d41586-021-00663-2. 33723408

[16] I. F. R. Oliveira , N. G. Pereira , L. F. Monteiro , et al., “Factors Influencing the Quality of Life and Mental Health of Brazilian Federal Education Network Employees: An Epidemiological Cross‐Sectional Study,” Heliyon 11, no. 3 (February 2025): e42029, 10.1016/j.heliyon.2025.e42029. 39981372 PMC11840539

[17] T. Vedoato , D. R. C. Pedro , M. J. Q. Galdino , et al., “Association Between Workaholism and Quality of Life in Stricto Sensu Graduate Professors in Nursing,” Revista Brasileira de Enfermagem 74, no. 2 (2021), 10.1590/0034-7167-2019-0901. 33886833

[18] A. Maqsood , S. Gul , N. Noureen , and A. Yaswi , “Dynamics of Perceived Stress, Stress Appraisal, and Coping Strategies in an Evolving Educational Landscape,” Behavioral Sciences 14, no. 7 (June 2024): 532, 10.3390/bs14070532. 39062355 PMC11274181

[19] C. M. Hazell , L. Chapman , S. F. Valeix , P. Roberts , J. E. Niven , and C. Berry , “Understanding the Mental Health of Doctoral Researchers: A Mixed Methods Systematic Review With Meta‐Analysis and Meta‐Synthesis,” Systematic Reviews 9, no. 1 (December 2020): 197, 10.1186/s13643-020-01443-1. 32847624 PMC7450565

[20] D. Trang , C. E. Swafford , T. A. Kreps , et al., “A Survey of the Severity of Mental Health Symptoms in the Planetary Science Community,” Nature Astronomy 8, no. 6 (June 2024): 691–696, 10.1038/s41550-024-02293-w.

[21] S. Guthrie , C. A. Lichten , J. Van Belle , S. Ball , A. Knack , and J. Hofman , “Understanding Mental Health in the Research Environment: A Rapid Evidence Assessment,” Rand Health Quarterly 7, no. 3 (2018): 2. 10.7249/rhq-07-03-02 PMC5873519 29607246

[22] F. Staniscuaski , L. Kmetzsch , R. C. Soletti , et al., “Gender, Race and Parenthood Impact Academic Productivity During the COVID‐19 Pandemic: From Survey to Action,” Frontiers in Psychology 12 (May 2021): 12, 10.3389/fpsyg.2021.663252. PMC8153681 34054667

[23] J. K. Hohls , H. H. König , E. Quirke , and A. Hajek , “Association Between Anxiety, Depression and Quality of Life: Study Protocol for a Systematic Review of Evidence From Longitudinal Studies,” BMJ Open 9, no. 3 (March 2019): e027218, 10.1136/bmjopen-2018-027218. PMC6429835 30837260

[24] A. P. Johnson and R. J. Lester , “Mental Health in Academia: Hacks for Cultivating and Sustaining Wellbeing,” American Journal of Human Biology 34, no. S1 (February 2022): e23664, 10.1002/ajhb.23664. 34357661

[25] C. Mendes‐Rodrigues , M. A. Ranal , and D. V. P. Carvalho , “Postgraduate Students: An Alert About Quality of Life,” World Journal of Education 9, no. 1 (February 2019): 135, 10.5430/wje.v9n1p135.

[26] O. Goñi‐Balentziaga , S. Vila , I. Ortega‐Saez , O. Vegas , and G. Azkona , “Professional Quality of Life in Research Involving Laboratory Animals,” Animals 11, no. 9 (September 2021): 2639, 10.3390/ani11092639. 34573605 PMC8465412

[27] L. Shamseer , D. Moher , M. Clarke , et al., “Preferred Reporting Items for Systematic Review and Meta‐Analysis Protocols (PRISMA‐P) 2015: Elaboration and Explanation,” BMJ 349, no. jan02 1 (January 2015): g7647, 10.1136/bmj.g7647. 25555855

[28] D. Moher , L. Shamseer , M. Clarke , et al., “Preferred Reporting Items for Systematic Review and Meta‐Analysis Protocols (PRISMA‐P) 2015 Statement,” Systematic Reviews 4, no. 1 (December 2015): 1, 10.1186/2046-4053-4-1. 25554246 PMC4320440

[29] A. Liberati , D. G. Altman , J. Tetzlaff , et al., “The PRISMA Statement for Reporting Systematic Reviews and Meta‐Analyses of Studies That Evaluate Healthcare Interventions: Explanation and Elaboration,” BMJ 339, no. jul21 1 (December 2009): b2700, 10.1136/bmj.b2700. 19622552 PMC2714672

[30] G. M. Tawfik , H. T. N. Giang , S. Ghozy , et al., “Protocol Registration Issues of Systematic Review and Meta‐Analysis Studies: A Survey of Global Researchers,” BMC Medical Research Methodology 20, no. 1 (December 2020): 213, 10.1186/s12874-020-01094-9. 32842968 PMC7448304

[31] A. Bannach‐Brown , T. Rackoll , N. Kaynak , et al., “Navigating PROSPERO4animals: 10 Top Tips for Efficient Pre‐Registration of Your Animal Systematic Review Protocol,” BMC Medical Research Methodology 24, no. 1 (January 2024): 20, 10.1186/s12874-024-02146-0. 38267888 PMC10807142

[32] R. T. Sataloff , M. L. Bush , R. Chandra , et al., “Systematic and Other Reviews: Criteria and Complexities,” Ear, Nose, & Throat Journal 100, no. 6 (July 2021): 403–406, 10.1177/01455613211025937. 34259592

[33] D. Moher , A. Liberati , J. Tetzlaff , and D. G. Altman , “Preferred Reporting Items for Systematic Reviews and Meta‐Analyses: The PRISMA Statement,” Journal of Clinical Epidemiology 62, no. 10 (October 2009): 1006–1012, 10.1016/j.jclinepi.2009.06.005. 19631508

[34] K. E. Hunter , A. C. Webster , M. J. Page , et al., “Searching Clinical Trials Registers: Guide for Systematic Reviewers,” BMJ 377 (April 2022): e068791, 10.1136/bmj-2021-068791. 35473822

[35] J. V. A. Franco , V. L. Garrote , C. M. Escobar Liquitay , and V. Vietto , “Identification of Problems in Search Strategies in Cochrane,” Research Synthesis Methods 9, no. 3 (September 2018): 408–416, 10.1002/jrsm.1302. 29761662

[36] E. Calderon Martinez , J. R. Flores Valdés , J. L. Castillo , et al., “10 Steps to Conduct a Systematic Review,” Cureus 15 (December 2023): e51422, 10.7759/cureus.51422. 38299136 PMC10828625

[37] A. Booth , “‘Brimful of STARLITE’”: Toward Standards for Reporting Literature Searches,” Journal of the Medical Library Association 94 (2006): 421, http://www.copernic. 17082834 PMC1629442

[38] H. Krumholz , “The Case for Duplication of Meta‐Analyses and Systematic Reviews,” BMJ 347 (2013): f5506, 10.1136/bmj.f5506. 24022045

[39] D. Moher , “The Problem of Duplicate Systematic Reviews,” BMJ 347 (2013): f5040, 10.1136/bmj.f5040. 23945367

[40] C. Lefebvre and S. Duffy , “Peer Review of Searches for Studies for Health Technology Assessments, Systematic Reviews, and Other Evidence Syntheses,” International Journal of Technology Assessment in Health Care 37, no. 1 (2021): e64, 10.1017/S0266462321000210. 34024305

[41] N. G. Pereira , R. M. F. Silva , I. F. R. Oliveira , et al., “Administrative Professionals' Quality of Life in Educational Institutions: A Systematic Review Protocol,” BMJ Open 13, no. 8 (August 2023): e074119, 10.1136/bmjopen-2023-074119. PMC10414068 37558456

[42] W. P. Costa , M. S. V. Fernandes , A. R. Memon , P. R. E. S. Noll , M. M. Sousa , and M. Noll , “Factors Influencing the Work of Researchers in Scientific Initiation: A Systematic Review Protocol,” PLoS One 19, no. 1 (January 2024): e0297186, 10.1371/journal.pone.0297186. 38295057 PMC10829991

[43] E. Dias , W. Pires da Costa , M. Da Silva Valadão Fernandes , S. N. Valente , P. Rayanne E. Silva Noll , and M. Noll , “Teachers' Quality of Life Perception During the COVID‐19 Pandemic: A Systematic Review Protocol,” Journal of Human Growth and Development 34, no. 2 (July 2024): 268–277, 10.36311/jhgd.v34.15837.

[44] M. B. Costa , R. M. F. Silva , K. V. C. Silva , et al., “Food Consumption and Mental Health in Children and Adolescents: A Systematic Review Protocol,” MethodsX 13 (December 2024): 103015, 10.1016/j.mex.2024.103015. 39583999 PMC11585736

[45] Y. Li and G. Zheng , “The Efficacy of Aquatic Therapy in Stroke Rehabilitation: A Protocol for Systematic Review and Meta‐Analysis,” Medicine 100, no. 48 (2021): e27825, 10.1097/MD.0000000000027825. 35049184 PMC9191287

[46] I. Pérez‐Neri , C. Pineda , J. L. Flores‐Guerrero , et al., “Adherence to Literature Search Reporting Guidelines in Leading Rheumatology Journals' Systematic Reviews: Umbrella Review Protocol,” Rheumatology International 42, no. 12 (August 2022): 2135–2140, 10.1007/s00296-022-05194-1. 36029320

[47] L. F. Terra , W. P. Costa , R. M. F. Silva , L. M. T. Rezende , M. Noll , and P. R. E. S. Noll , “Interventions Towards Barriers to the Practice of Physical Activity in Adolescence: A Systematic Review Protocol,” PLoS One 18, no. 7 (July 2023): e0287868, 10.1371/journal.pone.0287868. 37437054 PMC10337968

[48] M. Ouzzani , H. Hammady , Z. Fedorowicz , and A. Elmagarmid , “Rayyan—A Web and Mobile App for Systematic Reviews,” Systematic Reviews 5, no. 1 (December 2016): 210, 10.1186/s13643-016-0384-4. 27919275 PMC5139140

[49] M. Noll , N. Wedderkopp , C. R. Mendonça , and P. Kjaer , “Motor Performance and Back Pain in Children and Adolescents: A Systematic Review and Meta‐Analysis Protocol,” Systematic Reviews 9, no. 1 (December 2020): 212, 10.1186/s13643-020-01468-6. 32928303 PMC7491087

[50] M. J. Page , J. E. McKenzie , P. M. Bossuyt , et al., “The PRISMA 2020 Statement: An Updated Guideline for Reporting Systematic Reviews,” BMJ 372 (March 2021): n71, 10.1136/bmj.n71. 33782057 PMC8005924

[51] K. S. Tan , Y. C. Yeh , P. S. Adusumilli , and W. D. Travis , “Quantifying Interrater Agreement and Reliability Between Thoracic Pathologists: Paradoxical Behavior of Cohen's Kappa in the Presence of a High Prevalence of the Histopathologic Feature in Lung Cancer,” JTO Clinical and Research Reports 5, no. 1 (January 2024): 100618, 10.1016/j.jtocrr.2023.100618. 38283651 PMC10820331

[52] W. Vach and O. Gerke , “Gwet's AC1 Is Not a Substitute for Cohen's Kappa – A Comparison of Basic Properties,” MethodsX 10 (2023): 102212, 10.1016/j.mex.2023.102212. 37234937 PMC10205778

[53] A. Martín Andrés and M. Álvarez Hernández , “Hubert's Multi‐Rater Kappa Revisited,” British Journal of Mathematical and Statistical Psychology 73, no. 1 (February 2020): 1–22, 10.1111/bmsp.12167. 31056757

[54] B. M. Derksen , W. Bruinsma , J. C. Goslings , and N. W. L. Schep , “The Kappa Paradox Explained,” Journal of Hand Surgery 49, no. 5 (May 2024): 482–485, 10.1016/j.jhsa.2024.01.006. 38372689

[55] M. Delgado‐Rodríguez and M. Sillero‐Arenas , “Systematic Review and Meta‐Analysis,” Medicina Intensiva 42, no. 7 (October 2018): 444–453, 10.1016/j.medin.2017.10.003. 29169792

[56] M. Assel , D. Sjoberg , A. Elders , et al., “Guidelines for Reporting of Statistics for Clinical Research in Urology,” BJU International 123, no. 3 (March 2018): 401–410, 10.1111/bju.14640. PMC6397060 30537407

[57] M. M. Costa , M. M. Sousa , R. L. F. Coelho , M. M. Torres , and P. R. Silva , “From Ethical Conduct to Responsible Science in Management Research,” Revista de Administração de Empresas 65, no. 6 (2025), 10.1590/s0034-759020250610.

[58] B. Mullen , Advanced Basic Meta‐Analysis (Psychology Press, 2013), 10.4324/9780203771938.

[59] M. W. L. Cheung and R. Vijayakumar , “A Guide to Conducting a Meta‐Analysis,” Neuropsychology Review 26, no. 2 (June 2016): 121–128, 10.1007/s11065-016-9319-z. 27209412

[60] J. Gurevitch , J. Koricheva , S. Nakagawa , and G. Stewart , “Meta‐Analysis and the Science of Research Synthesis,” Nature 555, no. 7695 (March 2018): 175–182, 10.1038/nature25753. 29517004

[61] M. S. V. Fernandes , C. R. Mendonça , T. M. V. da Silva , P. R. S. Noll , L. C. de Abreu , and M. Noll , “Relationship Between Depression and Quality of Life Among Students: A Systematic Review and Meta‐Analysis,” Scientific Reports 13, no. 1 (April 2023): 6715, 10.1038/s41598-023-33584-3. 37185375 PMC10126541

[62] S. Shim , B. H. Yoon , I. S. Shin , and J. M. Bae , “Network Meta‐Analysis: Application and Practice Using Stata,” Epidemiology and Health 39 (October 2017): e2017047, 10.4178/epih.e2017047. 29092392 PMC5733388

[63] M. Ordak , “Implementation of SAMPL Guidelines: Recommendations for Improving Statistical Reporting in Biomedical Journals,” Clinical Medicine 25, no. 3 (May 2025): 100304, 10.1016/j.clinme.2025.100304. 40157607 PMC12036030

[64] T. A. Lang and D. G. Altman , “Basic Statistical Reporting for Articles Published in Biomedical Journals: The ‘Statistical Analyses and Methods in the Published Literature’ or the SAMPL Guidelines,” International Journal of Nursing Studies 52, no. 1 (January 2015): 5–9, 10.1016/j.ijnurstu.2014.09.006. 25441757

[65] H. J. Schünemann , W. Wiercioch , I. Etxeandia , et al., “Guidelines 2.0: Systematic Development of a Comprehensive Checklist for a Successful Guideline Enterprise,” Canadian Medical Association Journal 186, no. 3 (February 2014): E123–E142, 10.1503/cmaj.131237. 24344144 PMC3928232

[66] T. Piggott , R. L. Morgan , C. A. Cuello‐Garcia , et al., “Grading of Recommendations Assessment, Development, and Evaluations (GRADE) Notes: Extremely Serious, Grade's Terminology for Rating down by Three Levels,” Journal of Clinical Epidemiology 120 (April 2020): 116–120, 10.1016/j.jclinepi.2019.11.019. 31866468

[67] K. G. Bautista‐Orduno , E. G. Dorsey‐Trevino , J. G. Gonzalez‐Gonzalez , et al., “American Thyroid Association Guidelines Are Inconsistent With Grading of Recommendations Assessment, Development, and Evaluations—A Meta‐Epidemiologic Study,” Journal of Clinical Epidemiology 123 (July 2020): 180–188.e2, 10.1016/j.jclinepi.2020.02.010. 32145366

[68] S. H. Downs and N. Black , “The Feasibility of Creating a Checklist for the Assessment of the Methodological Quality Both of Randomised and Non‐Randomised Studies of Health Care Interventions,” Journal of Epidemiology and Community Health 52, no. 6 (June 1998): 377–384, 10.1136/jech.52.6.377. 9764259 PMC1756728

[69] B. J. Biggerstaff and D. Jackson , “The Exact Distribution of Cochran's Heterogeneity Statistic in One‐Way Random Effects Meta‐Analysis,” Statistics in Medicine 27, no. 29 (December 2008): 6093–6110, 10.1002/sim.3428. 18781561

[70] J. P. T. Higgins , J. Thomas , J. Chandler , et al., eds., Cochrane Handbook for Systematic Reviews of Interventions (Wiley, 2019), 10.1002/9781119536604.

[71] J. P. T. Higgins , S. G. Thompson , J. J. Deeks , and D. G. Altman , “Measuring Inconsistency in Meta‐Analyses,” BMJ 327, no. 7414 (September 2003): 557–560, 10.1136/bmj.327.7414.557. 12958120 PMC192859

[72] J. Bowden , G. Davey Smith , and S. Burgess , “Mendelian Randomization With Invalid Instruments: Effect Estimation and Bias Detection Through Egger Regression,” International Journal of Epidemiology 44, no. 2 (April 2015): 512–525, 10.1093/ije/dyv080. 26050253 PMC4469799

[73] N. Q. Ribeiro , C. R. de Mendonça , W. P. da Costa , et al., “Prevalence and Factors Associated With the Perpetration and Victimization of Teen Dating Violence: A Systematic Review and Meta‐Analysis Protocol,” MethodsX 13 (December 2024): 103003, 10.1016/j.mex.2024.103003. 39507383 PMC11538795

[74] J. I. F. Santos Jesus , M. Monfort‐Pañego , G. V. Alves Santos , et al., “Food, Quality of Life and Mental Health: A Cross‐Sectional Study With Federal Education Workers,” Nutrients 17, no. 15 (July 2025): 2519, 10.3390/nu17152519. 40806106 PMC12348065

[75] E. J. Andrews , S. Harper , T. Cashion , et al., “Supporting Early Career Researchers: Insights From Interdisciplinary Marine Scientists,” ICES Journal of Marine Science 77, no. 2 (March 2020): 476–485, 10.1093/icesjms/fsz247.

[76] A. Müller , “Mental Health Disorders: Prevalent but Widely Ignored in Academia?,” Journal of Physiology 598, no. 7 (April 2020): 1279–1281, 10.1113/JP279386. 32003870

[77] M. T. N. Noe , A. Masserey , A. Bober , S. T. Mol , and I. Guseva Canu , “Inventory of Mental Health Services in Academia and Researchers' Awareness of Their Availability: Mixed Method Research Protocol and Pilot Study in Switzerland,” International Journal of Public Health 70 (May 2025): 1607982, 10.3389/ijph.2025.1607982. 40470066 PMC12133474

[78] E. Cilli , J. Ranieri , F. Guerra , and D. Di Giacomo , “Early Career Researchers and Mental Health: Observational Study of Challenge and Wellbeing,” Health Science Reports 6, no. 11 (November 2023), 10.1002/hsr2.1649. PMC10661320 38028694

[79] H. Nicholls , D. Lamb , S. Johnson , P. Higgs , V. Pinfold , and J. Billings , “‘Fix the System…the People Who Are in It Are Not the Ones That Are Broken’ A Qualitative Study Exploring UK Academic Researchers' Views on Support at Work,” Heliyon 9, no. 10 (October 2023): e20454, 10.1016/j.heliyon.2023.e20454. 37860508 PMC10582291

[80] D. Johann , J. Neufeld , K. Thomas , J. Rathmann , and H. Rauhut , “The Impact of Researchers' Perceived Pressure on Their Publication Strategies,” Research Evaluation (March 2024), 10.1093/reseval/rvae011.

[81] R. S., “Publication Stress Amongst Scholars and Faculties: A Concern of Mental Health,” Mental Health and Social Inclusion 29, no. 5 (November 2025): 486–493, 10.1108/MHSI-10-2024-0177.

[82] J. E. González Flores , “Academic Fatigue of Young Researchers: The Price of the Publish or Perish Culture,” Cureus 17, no. 11 (November 2025): e97223, 10.7759/cureus.97223. 41426759 PMC12715826

[83] D. Ahmed , “From Curiosity to Contribution: Reflections of a Junior Researcher on His Journey Into Mental Health Research in Iraq,” Global Psychiatry Archives 6, no. 1 (April 2023): 1–5, 10.52095/gpa.2023.6210.1066.

