# Supplementary material for: Researchers' Mental Health and Quality of Life: A Protocol for Systematic Review and Meta‐Analysis
Source: Health Sci Rep. 2026 May 14;9(5):e72253. doi: 10.1002/hsr2.72253 (PMC13176651; doi:10.1002/hsr2.72253)
Supplement: Supplementary file 2 — Supplementary_file_S2_table_strings_in_databases_R2.docx. [file HSR2-9-e72253-s002.docx]

**FILE S2**

Details of Boolean search string for each database.

| CINAHL^®^ Plus via EBSCOhost^™^ interface | |
| --- | --- |
| **Blocks and Returns** | **Search strings** |
| #1  (Return: ‹*n*_1_›) | TI(researcher* OR scientist* OR professor* OR "postgraduate student*" OR lecturer* OR faculty) |
| #2  (Return: ‹*n*_2_›) | TI(associated OR "associated factor*" OR "associated factors" OR "correlated factor*" OR "related factor*" OR "risk factor*" OR "factors of risk" OR "individual factor*" OR "personal factor*" OR "internal factor*" OR "external factor*" OR "motivating factor*" OR motivational factor*") |
| #3  (Return: ‹*n*_3_›) | TI("quality of life" OR "life quality" OR "quality of working life" OR wellbeing OR "well-being" OR qol OR hqol OR hrqol OR wrqol OR whoqol OR "whoqol-bref" OR sf36 OR "sf-36" OR euroqol OR "eq-5d" OR "eq-5d-5l" OR "eq-5d-3l" OR qaly OR ocai OR msq OR copsoq OR"personal satisfaction") AND TI(anxiety OR angst OR hypervigilance OR ervousness OR anxiousness OR depression OR "depressive symptom*" OR "depressed mood" OR "mood disorder*" OR "major depressive disorder" OR "mental disorder*" OR "mental health" OR "mental hygiene" OR stress OR "psychological well-being" OR "mental well-being" OR "psychological problem*" OR "psychological health" OR "excessive worry*" OR gad-7 OR phq-9 OR phq OR mdi OR gad OR "ham-a" OR hama OR hads OR dass OR ghq-28) |
| #4  (Return: ‹*n*_4_›) | TI( “case series” OR “case‒control” OR “cohort” OR “correlational study” OR “cross-national” OR “cross-sectional” OR “demographic study” OR “descriptive study” OR “epidemiological follow-up” OR “epidemiological study ” OR “follow-up study” OR “incidence follow-up” OR “incidence study” OR “interview-based follow-up” OR “interview-based study” OR “longitudinal follow-up” OR “longitudinal observational” OR “longitudinal study” OR “nationally representative” OR “observational analysis” OR “observational data” OR “observational follow-up” OR “observational pilot” OR “observational study” OR “observational survey” OR “population-based” OR “prevalence study” OR “prospective follow-up” OR “prospective observational” OR “prospective study” OR “questionnaire-based follow-up” OR “questionnaire-based study” OR “registry-based follow-up” OR “registry-based study ” OR “retrospective follow-up” OR “retrospective observational” OR “retrospective study” OR survey OR “time-series study”) |
| #5  (Return: ‹*n_5_*›) | (#1) AND (#2) AND (#3) AND (#4) |
|  | **Return after refinement**:  ‹*N*› studies in the test carried out in ‹*date*›. |

| Embase^™^ via native interface | |
| --- | --- |
| **Blocks and Returns** | **Search strings** |
| #1  (Return: ‹*n*_1_›) | ti,ab,kw:(researcher* OR scientist* OR professor* OR lecturer* OR faculty OR "postgraduate student*") |
| #2  (Return: ‹*n*_2_›) | ti,ab,kw:(associated OR "associated factor*" OR "associated factors" OR "correlated factor*" OR "related factor*" OR "risk factor*" OR "factors of risk" OR "individual factor*" OR "personal factor*" OR "internal factor*" OR "external factor*" OR "motivating factor*" OR "motivational factor*") |
| #3  (Return: ‹*n*_3_›) | ti,ab,kw:("quality of life" OR "life quality" OR "quality of working life" OR wellbeing OR "well-being" OR qol OR hqol OR hrqol OR wrqol OR whoqol OR "whoqol-bref" OR sf36 OR "sf-36" OR euroqol OR "eq-5d" OR "eq-5d-5l" OR "eq-5d-3l" OR qaly OR ocai OR msq OR copsoq OR "personal satisfaction") AND ti,ab,kw:(anxiety OR angst OR hypervigilance OR nervousness OR anxiousness OR depression OR "depressive symptom*" OR "depressed mood" OR "mood disorder*" OR "major depressive disorder" OR "mental disorder*" OR mental health" OR "mental hygiene" OR stress OR "psychological well-being" OR "mental well-being" OR "psychological problem*" OR "psychological health" OR "excessive worry*" OR gad-7 OR phq-9 OR phq OR mdi OR gad OR "ham-a" OR hama OR hads OR dass OR ghq-28) |
| #4  (Return: ‹*n*_4_›) | ti,ab,kw:(“case series” OR “case‒control” OR “cohort” OR “correlational study” OR “cross-national” OR “cross-sectional” OR “demographic study” OR “descriptive study” OR “epidemiological follow-up” OR “epidemiological study ” OR “follow-up study” OR “incidence follow-up” OR “incidence study” OR “interview-based follow-up” OR “interview-based study” OR “longitudinal follow-up” OR “longitudinal observational” OR “longitudinal study” OR “nationally representative” OR “observational analysis” OR “observational data” OR “observational follow-up” OR “observational pilot” OR “observational study” OR “observational survey” OR “population-based” OR “prevalence study” OR “prospective follow-up” OR “prospective observational” OR “prospective study” OR “questionnaire-based follow-up” OR “questionnaire-based study” OR “registry-based follow-up” OR “registry-based study ” OR “retrospective follow-up” OR “retrospective observational” OR “retrospective study” OR survey OR “time-series study”) |
| #5  (Return: ‹*n_5_*›) | (#1) AND (#2) AND (#3) AND (#4) |
|  | **Return after refinement**:  ‹*N*› studies in the test carried out in ‹*date*›. |

| **LILACS via interface da Biblioteca Virtual em Saúde (BVS)** | |
| --- | --- |
| **Blocks and Returns** | **Search strings** |
| #1  (Return: ‹*n*_1_›) | (researcher* OR scientist* OR professor* OR "postgraduate student*" OR lecturer* OR faculty) |
| #2  (Return: ‹*n*_2_›) | (associated OR "associated factor*" OR "correlated factor*" OR "external factor*" OR "related factor*" OR "factor of risk" OR "risk factor*" OR "individual factor" OR "personal factor*" OR "internal factor" OR "motivating factor*" OR "motivation factor*" OR "motivational factor*" OR "factors of risk") |
| #3  (Return: ‹*n*_3_›) | ("life quality" OR "quality of life" OR "quality of working life" OR wellbeing OR "well-being" OR qol OR hqol OR hrqol OR "value of life" OR wrqol OR whoqol OR "whoqol-bref" OR "SF -36" OR SF36 OR “personal satisfaction” OR euroqol OR “eq-5d” OR “eq-5d-5 l” OR “eq-5d-3d” OR qaly OR ocai OR msq OR copsoq) AND  (anxiety OR angst OR hypervigilance OR nervousness OR anxiousness OR “C-DAS” OR “CFSS-DS” OR COHIP OR COHRQoL OR depression OR “depressive symptom*” OR “depressed mood” OR “mood disorder” OR “major depressive disorder” OR “mental disorder” OR “mental health” OR “mental hygiene” OR stress OR “psychological well-being” OR “mental well-being” OR “mental depressive disorders” OR “psychological problem” OR “psychological” OR “psychological health” OR “excessive worries” OR “excessive worry” OR “de disorder ” OR gad-7 OR phq-9 OR phq OR mdi OR GAD OR ham-a OR hama OR hads OR dass OR ghq-28) |
| #4  (Return: ‹*n*_4_›) | (“case series” OR “case control” OR “cohort” OR “correlational study” OR “cross-national” OR “cross-sectional” OR “demographic study” OR “descriptive study” OR “epidemiological follow-up” OR “epidemiological study” OR “follow-up study” OR “incidence follow-up” OR “incidence study” OR “interview-based follow-up” OR “interview-based study” OR “longitudinal follow-up” OR “longitudinal observational” OR “longitudinal study” OR “nationally representative” OR “observational analysis” OR “observational data” OR “observational follow-up” OR “observational pilot” OR “observational study” OR “observational survey” OR “population-based” OR “prevalence study” OR “prospective follow-up” OR “prospective observational” OR “prospective study” OR “questionnaire-based follow-up” OR “questionnaire-based study” OR “registry-based follow-up” OR “registry-based study” OR “retrospective follow-up” OR “retrospective observational” OR “retrospective study” OR survey OR “time-series study”) |
| #5  (Return: ‹*n_5_*›) | (#1) AND (#2) AND (#3) AND (#4) |
|  | **Return after refinement**:  ‹*N*› studies in the test carried out in ‹*date*›. |
|  |  |

| MEDLINE/PubMed^®^ via interface da National Library of Medicine^®^ (NLM^®^) | |
| --- | --- |
| **Blocks and Returns** | **Search strings** |
| #1  (Return: ‹*n*_1_›) | (researcher*[Title/Abstract] OR scientist*[Title/Abstract] OR professor*[Title/Abstract] OR "postgraduate student*"[Title/Abstract] OR lecture[Title/Abstract] OR faculty[Title/Abstract] OR “research personnel”[MeSH Terms] OR "faculty"[MeSH Terms]) |
| #2  (Return: ‹*n*_2_›) | (associated[Title/Abstract] OR "associated factor*"[Title/Abstract] OR "correlated factor*"[Title/Abstract] OR "external factor*"[Title/Abstract] OR "related factor*"[Title/Abstract] OR "factor of risk"[Title/Abstract] OR "risk factor*"[Title/Abstract] OR "individual factor"[Title/Abstract] OR "personal factor*"[Title/Abstract] OR " internal factor"[Title/Abstract] OR "motivating factor*"[Title/Abstract] OR "motivation factor*"[Title/Abstract] OR "motivational factor*"[Title/Abstract] OR "factors of risk"[Title/Abstract]) |
| #3  (Return: ‹*n*_3_›) | ("life quality"[Title/Abstract] OR "quality of life"[Title/Abstract] OR "quality of working life"[Title/Abstract] OR wellbeing[Title/Abstract] OR "well-being"[Title/Abstract] OR qol[Title/Abstract] OR hqol[Title/Abstract] OR hrqol[Title/Abstract] OR "value of life"[Title/Abstract] OR wrqol[Title/Abstract] OR whoqol[Title/Abstract] OR "whoqol-bref"[Title/Abstract] OR "SF -36"[Title/Abstract] OR SF36[Title/Abstract] OR "personal satisfaction"[Title/Abstract] OR euroqol[Title/Abstract] OR "eq-5d"[Title/Abstract] OR "eq-5d-5 l"[Title/Abstract] OR "eq-5d-3d"[Title/Abstract] OR qaly[Title/Abstract] OR ocai[Title/Abstract] OR msq[Title/Abstract] OR copsoq[Title/Abstract]) OR "quality of life"[MeSH Terms]) AND (anxiety[Title/Abstract] OR angst[Title/Abstract] OR hypervigilance[Title/Abstract] OR nervousness[Title/Abstract] OR anxiousness[Title/Abstract] OR "C-DAS"[Title/Abstract] OR "CFSS-DS"[Title/Abstract] OR COHIP[Title/Abstract] OR COHRQoL[Title/Abstract] OR depression[Title/Abstract] OR "depressive symptom*"[Title/Abstract] OR "depressed mood"[Title/Abstract] OR "mood disorder"[Title/Abstract] OR "major depressive disorder"[Title/Abstract] OR "mental disorder"[Title/Abstract] OR "mental health"[Title/Abstract] OR "mental hygiene"[Title/Abstract] OR stress[Title/Abstract] OR "psychological well-being"[Title/Abstract] OR "mental well-being"[Title/Abstract] OR "psychological problem"[Title/Abstract] OR "psychological"[Title/Abstract] OR "psychological health"[Title/Abstract] OR "excessive worries"[Title/Abstract] OR "excessive worry"[Title/Abstract] OR gad-7[Title/Abstract] OR phq-9[Title/Abstract] OR phq[Title/Abstract] OR mdi[Title/Abstract] OR GAD[Title/Abstract] OR ham-a[Title/Abstract] OR hama[Title/Abstract] OR hads[Title/Abstract] OR dass[Title/Abstract] OR ghq-28[Title/Abstract] OR "Mental Health"[MeSH Terms] OR "Anxiety Disorders"[MeSH Terms] OR "Depression"[MeSH Terms] OR "Stress, Psychological"[MeSH Terms]) |
| #4  (Return: ‹*n*_4_›) | ("case series"[Title/Abstract] OR "case‒control"[Title/Abstract] OR "cohort"[Title/Abstract] OR "correlational study"[Title/Abstract] OR "cross-national"[Title/Abstract] OR "cross-sectional"[Title/Abstract] OR "demographic study"[Title/Abstract] OR "descriptive study"[Title/Abstract] OR "epidemiological follow-up"[Title/Abstract] OR "epidemiological study "[Title/Abstract] OR "follow-up study"[Title/Abstract] OR "incidence follow-up"[Title/Abstract] OR "incidence study"[Title/Abstract] OR "interview-based follow-up"[Title/Abstract] OR "interview-based study"[Title/Abstract] OR "longitudinal follow-up"[Title/Abstract] OR "longitudinal observational"[Title/Abstract] OR "longitudinal study"[Title/Abstract] OR "nationally representative"[Title/Abstract] OR "observational analysis"[Title/Abstract] OR "observational data"[Title/Abstract] OR "observational follow-up"[Title/Abstract] OR "observational pilot"[Title/Abstract] OR "observational study"[Title/Abstract] OR "observational survey"[Title/Abstract] OR "population-based"[Title/Abstract] OR "prevalence study"[Title/Abstract] OR "prospective follow-up"[Title/Abstract] OR "prospective observational"[Title/Abstract] OR "prospective study"[Title/Abstract] OR "questionnaire-based follow-up"[Title/Abstract] OR "questionnaire-based study"[Title/Abstract] OR "registry-based follow-up"[Title/Abstract] OR "registry-based study "[Title/Abstract] OR "retrospective follow-up"[Title/Abstract] OR "retrospective observational"[Title/Abstract] OR "retrospective study"[Title/Abstract] OR survey[Title/Abstract] OR "time-series study"[Title/Abstract] OR "Epidemiologic Studies"[MeSH Terms] OR "Cross-Sectional Studies"[MeSH Terms] OR "Case-Control Studies"[MeSH Terms] |
| #5  (Return: ‹*n_5_*›) | (#1) AND (#2) AND (#3) AND (#4) |
|  | **Return after refinement**:  ‹*N*› studies in the test carried out in ‹*date*›. |

Note: MeSH is acronymous of Medical Subject Headings.

| Scopus^™^ via native interface | |
| --- | --- |
| **Blocks and Returns** | **Search strings** |
| #1  (Return: ‹*n*_1_›) | TITLE-ABS-KEY ( researcher* OR scientist* OR professor* OR "postgraduate student*" OR lecturer* OR faculty ) |
| #2  (Return: ‹*n*_2_›) | TITLE-ABS-KEY ( associated OR "associated factor*" OR "correlated factor*" OR "external factor*" OR "related factor*" OR "factor of risk" OR "risk factor*" OR "individual factor" OR "personal factor*" OR "internal factor" OR "motivating factor*" OR "motivation factor*" OR "motivational factor*" OR "factors of risk" ) |
| #3  (Return: ‹*n*_3_›) | TITLE-ABS-KEY ( ( "life quality" OR "quality of life" OR "quality of working life" OR wellbeing OR "well-being" OR qol OR hqol OR hrqol OR "value of life" OR wrqol OR whoqol OR "whoqol-bref" OR "SF -36" OR SF36 OR "personal satisfaction" OR euroqol OR "eq-5d" OR "eq-5d-5 l" OR "eq-5d-3d" OR qaly OR ocai OR msq OR copsoq ) AND ( anxiety OR angst OR hypervigilance OR nervousness OR anxiousness OR "C-DAS" OR "CFSS-DS" OR COHIP OR COHRQoL OR depression OR "depressive symptom*" OR "depressed mood" OR "mood disorder" OR "major depressive disorder" OR "mental disorder" OR "mental health" OR "mental hygiene" OR stress OR "psychological well-being" OR "mental well-being" OR "mental depressive disorders" OR "psychological problem" OR "psychological" OR "psychological health" OR "excessive worries" OR "excessive worry" OR "de disorder " OR gad-7 OR phq-9 OR phq OR mdi OR GAD OR ham-a OR hama OR hads OR dass OR ghq-28 ) ) |
| #4  (Return: ‹*n*_4_›) | TITLE-ABS-KEY ( “case series” OR “case control” OR “cohort” OR “correlational study” OR “cross-national” OR “cross-sectional” OR “demographic study” OR “descriptive study” OR “epidemiological follow-up” OR “epidemiological study” OR “follow-up study” OR “incidence follow-up” OR “incidence study” OR “interview-based follow-up” OR “interview-based study” OR “longitudinal follow-up” OR “longitudinal observational” OR “longitudinal study” OR “nationally representative” OR “observational analysis” OR “observational data” OR “observational follow-up” OR “observational pilot” OR “observational study” OR “observational survey” OR “population-based” OR “prevalence study” OR “prospective follow-up” OR “prospective observational” OR “prospective study” OR “questionnaire-based follow-up” OR “questionnaire-based study” OR “registry-based follow-up” OR “registry-based study” OR “retrospective follow-up” OR “retrospective observational” OR “retrospective study” OR survey OR “time-series study” ) |
| #5  (Return: ‹*n_5_*›) | (#1) AND (#2) AND (#3) AND (#4) |
|  | **Return after refinement**:  ‹*N*› studies in the test carried out in ‹*date*›. |

| Web of Science^™^ via Clarivate | |
| --- | --- |
| **Blocks and Returns** | **Search strings** |
| #1  (Return: ‹*n*_1_›) | TS=(researcher* OR scientist* OR professor* OR "postgraduate student*" OR lecturer* OR faculty ) |
| #2  (Return: ‹*n*_2_›) | TS=(associated OR "associated factor*" OR "correlated factor*" OR "external factor*" OR "related factor*" OR "factor of risk" OR "risk factor*" OR "individual factor" OR "personal factor*" OR "internal factor" OR "motivating factor*" OR "motivation factor*" OR "motivational factor*" OR "factors of risk" ) |
| #3  (Return: ‹*n*_3_›) | TS=(("life quality" OR "quality of life" OR "quality of working life" OR wellbeing OR "well-being" OR qol OR hqol OR hrqol OR "value of life" OR wrqol OR whoqol OR "whoqol-bref" OR "SF -36" OR SF36 OR “personal satisfaction” OR euroqol OR “eq-5d” OR “eq-5d-5 l” OR “eq-5d-3d” OR qaly OR ocai OR msq OR copsoq) AND  (anxiety OR angst OR hypervigilance OR nervousness OR anxiousness OR “C-DAS” OR “CFSS-DS” OR COHIP OR COHRQoL OR depression OR “depressive symptom*” OR “depressed mood” OR “mood disorder” OR “major depressive disorder” OR “mental disorder” OR “mental health” OR “mental hygiene” OR stress OR “psychological well-being” OR “mental well-being” OR “mental depressive disorders” OR “psychological problem” OR “psychological” OR “psychological health” OR “excessive worries” OR “excessive worry” OR “de disorder ” OR gad-7 OR phq-9 OR phq OR mdi OR GAD OR ham-a OR hama OR hads OR dass OR ghq-28)) |
| #4  (Return: ‹*n*_4_›) | TS=(“case series” OR “case control” OR “cohort” OR “correlational study” OR “cross-national” OR “cross-sectional” OR “demographic study” OR “descriptive study” OR “epidemiological follow-up” OR “epidemiological study” OR “follow-up study” OR “incidence follow-up” OR “incidence study” OR “interview-based follow-up” OR “interview-based study” OR “longitudinal follow-up” OR “longitudinal observational” OR “longitudinal study” OR “nationally representative” OR “observational analysis” OR “observational data” OR “observational follow-up” OR “observational pilot” OR “observational study” OR “observational survey” OR “population-based” OR “prevalence study” OR “prospective follow-up” OR “prospective observational” OR “prospective study” OR “questionnaire-based follow-up” OR “questionnaire-based study” OR “registry-based follow-up” OR “registry-based study” OR “retrospective follow-up” OR “retrospective observational” OR “retrospective study” OR survey OR “time-series study”) |
| #5  (Return: ‹*n_5_*›) | (#1) AND (#2) AND (#3) AND (#4) |
|  | **Return after refinement**:  ‹*N*› studies in the test carried out in ‹*date*›. |

| PsycArticles via interface PsycNet | |
| --- | --- |
| **Blocks and Returns** | **Search strings** |
| #1  (Return: ‹*n*_1_›) | **Any Field**: researcher* *OR* **Any Field**: scientist* *OR* **Any Field**: professor* *OR* **Any Field**: "postgraduate student*" *OR* **Any Field**: lecturer* *OR* **Any Field**: faculty |
| #2  (Return: ‹*n*_2_›) | **Any Field**: associated *OR* **Any Field**: "associated factor*" *OR* **Any Field**: "correlated factor*" *OR* **Any Field**: "external factor*" *OR* **Any Field**: "related factor*" *OR* **Any Field**: "factor of risk" *OR* **Any Field**: "risk factor*" *OR* **Any Field**: "individual factor" *OR* **Any Field**: "personal factor*" *OR* **Any Field**: "internal factor" *OR* **Any Field**: "motivating factor*" *OR* **Any Field**: "motivation factor*" *OR* **Any Field**: "motivational factor*" *OR* **Any Field**: "factors of risk" |
| #3  (Return: ‹*n*_3_›) | **Any Field**: ("life quality" OR "quality of life" OR "quality of working life" OR wellbeing OR "well-being" OR qol OR hqol OR hrqol OR "value of life" OR wrqol OR whoqol OR "whoqol-bref" OR "SF -36" OR SF36 OR “personal satisfaction” OR euroqol OR “eq-5d” OR “eq-5d-5 l” OR “eq-5d-3d” OR qaly OR ocai OR msq OR copsoq) AND  (anxiety OR angst OR hypervigilance OR nervousness OR anxiousness OR “C-DAS” OR “CFSS-DS” OR COHIP OR COHRQoL OR depression OR “depressive symptom*” OR “depressed mood” OR “mood disorder” OR “major depressive disorder” OR “mental disorder” OR “mental health” OR “mental hygiene” OR stress OR “psychological well-being” OR “mental well-being” OR “mental depressive disorders” OR “psychological problem” OR “psychological” OR “psychological health” OR “excessive worries” OR “excessive worry” OR “de disorder ” OR gad-7 OR phq-9 OR phq OR mdi OR GAD OR ham-a OR hama OR hads OR dass OR ghq-28) |
| #4  (Return: ‹*n*_4_›) | **Any Field**: “case series” OR “case control” OR “cohort” OR “correlational study” OR “cross-national” OR “cross-sectional” OR “demographic study” OR “descriptive study” OR “epidemiological follow-up” OR “epidemiological study” OR “follow-up study” OR “incidence follow-up” OR “incidence study” OR “interview-based follow-up” OR “interview-based study” OR “longitudinal follow-up” OR “longitudinal observational” OR “longitudinal study” OR “nationally representative” OR “observational analysis” OR “observational data” OR “observational follow-up” OR “observational pilot” OR “observational study” OR “observational survey” OR “population-based” OR “prevalence study” OR “prospective follow-up” OR “prospective observational” OR “prospective study” OR “questionnaire-based follow-up” OR “questionnaire-based study” OR “registry-based follow-up” OR “registry-based study” OR “retrospective follow-up” OR “retrospective observational” OR “retrospective study” OR survey OR “time-series study” |
| #5  (Return: ‹*n_5_*›) | (#1) AND (#2) AND (#3) AND (#4) |
|  | **Return after refinement**:  ‹*N*› studies in the test carried out in ‹*date*›. |
