# Supplementary material for: Researchers' Mental Health and Quality of Life: A Protocol for Systematic Review and Meta‐Analysis
Source: Health Sci Rep. 2026 May 14;9(5):e72253. doi: 10.1002/hsr2.72253 (PMC13176651; doi:10.1002/hsr2.72253)
Supplement: Supplementary file 3 — Supplementary_file_S3_table_included_in_the_SR_R2.docx. [file HSR2-9-e72253-s001.docx]

**File S3**

Characteristics, methodological features, and main outcomes of the studies included in the systematic review.

| **#** | **First author** | **Year/ Country** | **Study design** | **Population / Academic role** | **Sample size** | **Age range (years)** | **Instrument(s)** | **Outcome(s) assessed** | **Associated factors** | **Effect size (95% CI)** | **Statistical test** | **P-value** |
| --- | --- | --- | --- | --- | --- | --- | --- | --- | --- | --- | --- | --- |
| 1 | ---- | ----- | ---- | ---- | ---- | ---- | --- | --- | --- | --- | ---- |  |
|  |  |  |  |  |  |  |  |  |  |  |  |  |
|  |  |  |  |  |  |  |  |  |  |  |  |  |
|  |  |  |  |  |  |  |  |  |  |  |  |  |
